# Changing sperm donors—a shortcut to pregnancy or just a myth?

**DOI:** 10.1007/s10815-025-03464-y

**Published:** 2025-03-31

**Authors:** Shimi Barda, Yael Eliner, Noga Fuchs Weizman, Hadar Amir, Sandra E. Kleiman, Foad Azem, Ron Hauser

**Affiliations:** 1 The Institute for the Study of Fertility and Racine IVF Unit, Lis Maternity Hospital, Tel Aviv Sourasky Medical Center, 6 Weizman Street, 6423906 Tel Aviv, Israel; 2 Faculty of Medical & Health Sciences, Tel Aviv University, Tel Aviv, Israel; 3 Israel Academic College in Ramat Gan, Ramat Gan, Israel; 4 Department of Obstetrics and Gynecology, Zucker School of Medicine at Hofstra/Northwell, Lenox Hill Hospital, New York, NY USA

**Keywords:** Intrauterine insemination (IUI), Sperm donor replacement, Assisted reproductive technology (ART), Ovulation induction, Pregnancy success rates

## Abstract

**Purpose:**

Changing sperm donors after unsuccessful intrauterine insemination (IUI) cycles is a common yet understudied practice. This study evaluates whether switching sperm donors impacts the number of IUI cycles required to achieve pregnancy.

**Methods:**

This retrospective cohort study analyzed 312 women undergoing donor sperm IUI at Lis Maternity Hospital, Tel Aviv Sourasky Medical Center, from 1992 to 2020. Participants were divided into two groups: Group A (conceived using only one donor) and Group B (switched donors after initial unsuccessful attempts). The primary outcome was the number of IUI cycles until pregnancy. Statistical analyses included *t*-tests, ANOVA, and multivariate analysis of covariance (MANCOVA).

**Results:**

Women in Group A required fewer cycles (mean 3.78 ± 1.90) to achieve pregnancy compared to Group B (mean 6.07 ± 2.95, *P* < .001). However, after switching donors, the mean number of cycles needed in Group B (2.23 ± 1.61) was significantly lower than the total cycles required by Group A (*P* < .001). Cumulative live birth rates were higher in Group A (50.5% after three cycles; 81.5% after six cycles) compared to Group B (26.0% after three cycles; 61.9% after six cycles).

**Conclusion:**

Switching sperm donors after repeated unsuccessful IUI attempts significantly reduces the additional number of cycles needed to achieve pregnancy. These findings suggest that sperm-oocyte compatibility may influence IUI success. Clinicians should consider donor replacement after multiple failures. Prospective studies are required to confirm these results and investigate underlying biological mechanisms.

## Introduction

Advances in sperm preservation and the establishment of sperm banks over the past several decades have significantly increased the utilization of intrauterine insemination (IUI) with donor sperm, particularly among single women, lesbian couples, and heterosexual couples experiencing male infertility [1–3]. These advancements have transformed reproductive options, making donor sperm a critical resource in assisted reproductive technologies.

To prevent the transmission of sexually transmitted infections (STIs) such as HIV and hepatitis B and C, donor sperm must undergo cryopreservation and quarantine for at least 6 months [4]. However, the cryopreservation process often negatively affects sperm motility and viability [5]. To address these challenges, sperm banks adhere to strict quality standards, ensuring that vials for IUI contain a minimum of 15–20 million motile spermatozoa with progressive motility [6, 7].

The success rates of IUI are influenced by various factors, including the duration of infertility [8], ovarian stimulation protocols [9–11], and endometrial thickness at ovulation [12]. Among these, one of the most significant predictors of success is the woman’s age [13–16]. While the pregnancy rate per IUI cycle is approximately 16.4% [17], cumulative clinical pregnancy rates can reach 61–77% after three to four cycles [18]. Given the cost-effectiveness and minimal invasiveness of IUI, it is often recommended as an initial treatment before transitioning to in vitro fertilization (IVF) [19, 20].

Despite these advancements, some patients and practitioners consider changing the sperm donor after several unsuccessful IUI cycles, assuming that it might improve the chances of conception. This decision is often influenced by the ease of switching donors compared to adjusting other treatment parameters and by the perception that a different donor may yield better results. While this approach has not been systematically evaluated in controlled settings, it is commonly discussed in clinical practice and online forums. Notably, safety considerations are important when contemplating donor replacement. Research in natural conception has demonstrated that changing partners is associated with increased preeclampsia risk [21, 22], potentially due to insufficient maternal immunological adaptation to paternal antigens [23]. Whether similar mechanisms apply in donor sperm replacement remains understudied.

This study aims to address the clinical knowledge gap by evaluating whether replacing a sperm donor affects the number of IUI cycles required to achieve pregnancy and by exploring the potential interplay between donor replacement and ovulation induction protocols. By focusing on clinical outcomes, this study seeks to clarify whether donor replacement serves as a viable intervention to optimize success rates in assisted reproduction.

## Materials and methods

### Study design

This retrospective study utilized data from women who conceived for the first time through artificial insemination with donor sperm at the Institute for the Study of Fertility, Lis Maternity Hospital, Tel Aviv Sourasky Medical Center, between 1992 and 2020. The study was approved by the Institutional Review Board (IRB), which granted a waiver for informed consent. Women were classified into two groups: Group A included women who conceived after using a single sperm donor, while Group B comprised women who failed to conceive with their first donor but achieved pregnancy after switching to a second donor. Each group was further divided into three subgroups based on the ovulation induction method associated with successful conception: Group N (natural cycles), Group CC (at least one cycle of clomiphene citrate), and Group GF (at least one cycle of gonadotropins). This stratification allowed for a nuanced comparison of outcomes across different ovulation induction methods.

### Study population

The study included women meeting the inclusion criteria who underwent hormonal profile assessments on day 3 of their menstrual cycle and gynecological ultrasound scans. Inclusion criteria required a normal basal hormonal profile, including follicle-stimulating hormone (FSH), luteinizing hormone (LH), thyroid-stimulating hormone (TSH), testosterone, and prolactin levels. Data collected included age, body mass index (BMI), number of intrauterine insemination (IUI) cycles, and ovarian stimulation protocols. To isolate the specific outcomes of repeated IUI attempts, women who conceived after a single treatment cycle or who subsequently underwent in vitro fertilization (IVF) were excluded. All sperm donors whose samples were used in this study had a history of previously reported pregnancies.

### Follicular monitoring and ovulation induction protocols

All women initially underwent IUI treatments during natural cycles. If pregnancy was not achieved after two to three cycles, controlled ovarian hyperstimulation was recommended. The first-line treatment consisted of clomiphene citrate (CC) administered as a 5-day course of 100 mg daily. If pregnancy was still not achieved after two to three additional cycles, treatment with low-dose gonadotropins (GF) was recommended. Women aged ≥ 40 years were recommended to begin with at least one natural cycle and, if necessary, proceed directly to GF therapy. Stimulation was monitored through transvaginal ultrasound (TVUS) to assess follicular number and diameter, as well as blood levels of estradiol and progesterone. When the leading follicle reached a mean diameter of 18 mm and endometrial thickness was at least 6 mm as determined by TVUS, either 10,000 IU of human chorionic gonadotropin (hCG) or 250 mcg of Ovitrelle was administered. IUI was scheduled for 36 h post-administration.

### Sperm samples

Semen samples were obtained from healthy donors who underwent comprehensive physical examinations and serological screening prior to acceptance into the donor program. Each ejaculate was liquefied for at least 30 min at 37 °C and analyzed for semen volume, sperm concentration, and motility percentage. Morphologically normal sperm were assessed on Papanicolaou-stained smears using strict criteria [24–26]. The samples were processed as follows: they were washed with human tubal fluid medium (Irvine Scientific, Santa Ana, CA, USA) supplemented with 1% human serum albumin (Kamapharm Human Albumin; Kamada, Kibbutz Beit Kama, Israel). The washed sperm was then carefully diluted with an equal volume of freezing medium containing test yolk buffer (Irvine Scientific). Following dilution, the mixture was equilibrated at room temperature for 15 min, sealed in 0.5-ml straws, and gradually cooled using a semi-programmable freezer (Nicool LM-10; Air Liquid, Paris, France). Finally, the samples were transferred to liquid nitrogen (− 196 °C) for storage. Prior to insemination, the samples were thawed at 37 °C for 10 min and evaluated for total motile sperm count. Only samples containing 15–20 million motile spermatozoa post-thaw were used for insemination. The laboratory participates in international quality control programs (UK NEQAS, External Quality Assessment Schemes) to ensure reliability in sperm concentration, motility, and morphology evaluations.

### Statistics

Statistical analyses were conducted using SPSS for Windows, version 22.0. A two-tailed *P*-value < 0.05 was considered statistically significant. Descriptive statistics were expressed as mean ± standard deviation. Comparisons of continuous variables between two groups were conducted using the Student’s *t*-test or the nonparametric Wilcoxon test for non-normally distributed data. For comparisons among three groups, analysis of variance (ANOVA) with Dunnett’s multiple comparison procedure or the nonparametric Kruskal–Wallis test was used. The Pearson correlation coefficient was employed to assess the relationship between two continuous variables, while associations between categorical variables were evaluated using the chi-square test. The effect of multiple continuous and categorical variables on continuous-dependent variables was analyzed using multivariate analysis of covariance (MANCOVA).

## Results

The impact of sperm donor replacement on the number of cycles required to achieve pregnancy was assessed in 312 women who underwent a total of 1339 IUI cycles. Of these, 242 women were in Group A, and 70 women were in Group B. The mean ages of the women in Groups A and B were similar (37.03 ± 3.58 and 37.26 ± 3.28 years, respectively; *P* = 0.63). Similarly, the mean follicle-stimulating hormone (FSH) plasma concentrations on the third day of the menstrual cycle were comparable between Group A (6.43 ± 2.52 IU/L) and Group B (6.17 ± 2.67 IU/L; *P* = 0.51).

### Number of IUI cycles needed for pregnancy achievement

Women in Group A required a mean of 3.78 ± 1.90 IUI cycles to achieve pregnancy, compared to 6.07 ± 2.95 cycles in Group B (*P* < 0.001). Notably, the number of IUI cycles completed with the first donor in Group B (3.84 ± 2.48 cycles) was comparable to the total cycles in Group A (3.78 ± 1.90 cycles; *P* = 0.837). However, comparing the mean number of IUI cycles completed by Group A women to the cycles with the second donor in Group B (2.23 ± 1.61) revealed a significant advantage for the second donor in Group B (*P* < 0.001).

### Comparisons of ovulatory induction protocols

The mean number of IUI cycles required to achieve pregnancy was compared among three subgroups based on the final ovulatory induction protocol associated with successful conception. Women in Subgroup N (natural cycles) required a mean of 2.77 ± 0.98 cycles, Subgroup CC (clomiphene citrate) required 4.27 ± 2.04 cycles, and Subgroup GF (gonadotropins) required 5.64 ± 2.61 cycles (*P* < 0.001). The mean ages of women in these subgroups were similar (36.2 ± 3.62, 36.7 ± 2.89, and 37.3 ± 3.61 years, respectively; *P* = 0.69).

Further analyses compared the mean number of IUI cycles in Group A with the following: (1) the total IUI cycles in Group B, (2) the cycles with the first donor in Group B, (3) the cycles with the second donor in Group B, and (4) a direct comparison between the first and second donor cycles in Group B. The detailed results of these comparisons are presented in Table 1.

**Table 1:** Comparison of IUI cycles required for pregnancy by ovulation induction protocols

Treatment subgroup	IUI cycles in Group A	IUI cycles in Group B	IUI cycles in Group B (1st donor)	IUI cycles in Group B (2nd donor)
N (*n*)	2.72 ± 0.97^b,c^ (93)	3.17 ± 1.03 (13)	2.00 ± 1.04^b,d^	1.17 ± 0.39^c,d^
CC (*n*)	3.79 ± 1.78^a,c^ (67)	5.32 ± 2.54^a^ (19)	3.10 ± 2.05	2.21 ± 1.78^c^
GF (*n*)	4.82 ± 2.10^a,c^ (82)	7.42 ± 2.78^a^ (38)	4.63 ± 2.45^d^	2.60 ± 1.64^c,d^

The same superscript letter (a, b, c, d) within a row indicates a significant difference between groups (*P* < .05). *N*, natural cycles; *CC*, clomiphene citrate; *GF*, gonadotropins. Values are expressed as mean ± standard deviation unless otherwise specified

### Percentages of treatment cycles according to study groups

The proportions of natural cycles and hormone therapy cycles (CC or GF) were analyzed between Groups A and B. While natural cycles were more common in Group A, hormone therapy cycles (CC or GF) were significantly more frequent in Group B (*P* < 0.001; Table 2).

**Table 2:** Distribution of hormone therapy cycles by study groups

	**% of *N* cycles**	**% of CC cycles**	**% of GF cycles**
Group A	66.95 ± 32.98	22 ± 28.90	11.05 ± 20.09
Group B	53.99 ± 30.68	31.22 ± 26.67	14.79 ± 17.22
*P* value	.003	.004	.008

*N*, natural cycles; *CC*, clomiphene citrate; *GF*, gonadotropins

### Cumulative live birth rate analysis

The cumulative live birth rate (CLBR) improved progressively with additional treatment cycles, reaching 49.1% after three cycles and 78.9% after six cycles in the overall population (Table 3). Age stratification revealed significant variations, with younger women achieving higher CLBR. Comparison between treatment groups indicated that women in Group A (single donor) demonstrated significantly higher CLBR compared to women in Group B (donor replacement) after both three (50.5% vs. 26.0%) and six cycles (81.5% vs. 61.9%). Accordingly, women in Group A required fewer cycles to achieve pregnancy compared to women in Group B (3.78 ± 1.90 vs. 6.07 ± 2.95, respectively; *P* < 0.001). Notably, in Group B, switching to a second donor significantly reduced the number of cycles needed to achieve pregnancy (2.23 ± 1.61 vs. 3.84 ± 2.48 with the first donor).

**Table 3:** Cumulative live birth rate (CLBR) analysis

Group/age	CLBR after 3 cycles (%)	CLBR after 6 cycles (%)
Overall	49.1	78.9
< 30 yrs	65.4	90.2
30–34 yrs	55.4	84.1
35–39 yrs	44.3	73.4
≥ 40 yrs	30.7	62.7
Group A	50.5	81.5
Group B	26.0	61.9

### The effect of additional variables on the number of IUI cycles required for pregnancy achievement

The relationship between continuous variables affecting pregnancy success and the number of IUI cycles required was analyzed using Pearson’s correlation coefficient. Significant correlations were observed with blood estrogen levels (*P* < 0.001), endometrial thickness (*P* < 0.05), and the number of mature follicles (*P* < 0.001) on the day of hCG administration.

A multivariate analysis of covariance (MANCOVA) was conducted to assess the association of these variables with the study groups (A and B) and treatment subgroups (N, CC, and GF). This analysis identified a significant association between the treatment subgroups and the number of IUI cycles required to achieve pregnancy (*P* < 0.001). However, no significant associations were observed for blood estrogen levels, endometrial thickness, or the number of mature follicles on the day of hCG administration.

## Discussion

This study systematically evaluates the impact of sperm donor replacement on the number of IUI cycles required to achieve pregnancy. The results suggest that replacing the sperm donor after repeated unsuccessful attempts can significantly reduce the number of additional cycles needed to conceive. Specifically, while women in Group A required fewer overall cycles to achieve pregnancy, those in Group B demonstrated improved outcomes with their second donor, requiring significantly fewer cycles compared to their first donor.

### Interpretation of results

To further assess potential biases and confounding factors influencing the number of IUI cycles required for pregnancy, we performed a pairwise correlation analysis. This analysis revealed significant associations between the number of cycles and recognized predictors of pregnancy success, including blood estrogen levels, endometrial thickness, and the number of mature follicles on the day of hCG administration [27, 28], Interestingly, there was no significant correlation between the woman’s age and successful pregnancy achievement, likely due to the similar age distribution between groups A and B, as well as within their hormonal treatment subgroups. These findings reinforce the validity of our group comparisons and suggest that other factors, such as hormonal response and donor replacement, were more influential in determining outcomes. The cumulative live birth rate analysis further supports these findings, showing different trajectories for patients who remained with a single donor versus those who switched donors. While this data reinforces the value of persisting with treatment over multiple cycles, it also highlights the potential benefit of donor replacement in cases of repeated failure.

The observed reduction in cycles with the second donor in Group B supports the hypothesis that sperm-oocyte incompatibility may have contributed to initial treatment failures. This aligns with prior studies indicating that genetic [29, 30] and immunological factors [31] play a role in fertilization success and embryo development. Donor replacement might overcome these barriers, improving fertilization rates and pregnancy outcomes.

The greater number of hormone-stimulated cycles in Group B also likely contributed to improved outcomes. Hormonal stimulation enhances IUI success by increasing the number of mature follicles and improving endometrial receptivity [9, 32]. This highlights the importance of tailoring ovulation induction protocols based on patient response. However, the observed differences in outcomes between natural cycles and hormone-stimulated cycles could complicate the conclusions about donor replacement, as these factors are intertwined and may independently influence success rates. Future studies should stratify groups based on the type and number of hormonal treatments received before and after donor replacement to more precisely assess the individual contributions of these variables to treatment outcomes.

Division of the women into subgroups according to treatment protocols revealed that the number of IUI cycles required to achieve pregnancy increased according to the protocol that had been followed (N < CC < GF).

This division into treatment subgroups, however, did not address the question of whether a woman who replaced a donor underwent the same hormonal treatment that she received with the first donor. In fact, there is a greater chance that the Group B women underwent most of their natural treatments with the first donor and that most of their treatments with the second donor included ovulation induction. as demonstrated by the treatment cycle distribution shown in the results section.

Nevertheless, the cumulative pregnancy rate increases over multiple cycles, with up to 61.15% success after three cycles [18]. This gradual increase weakens the direct association between donor replacement and reduced IUI cycles. It is likely that the observed differences are influenced not only by donor replacement but also by the cumulative effect of multiple treatment cycles.

### Clinical implications

These findings underscore the importance of reassessing donor compatibility in cases of repeated IUI failures. Patients who experience unsuccessful cycles should be counseled about the potential benefits of donor replacement, particularly when sperm-oocyte interaction issues are suspected. Optimizing ovulation induction protocols in parallel may further enhance success rates and minimize patient burden. When counseling patients about treatment options, clinicians can now utilize the cumulative live birth rate data to provide more accurate prognostic information. The significant reduction in cycles needed with the second donor suggests that donor replacement can be an effective strategy after multiple failures. However, clinicians should also consider safety aspects, as research in natural conception suggests that changing partners might be associated with increased preeclampsia risk [21–23]

Patients often consider changing sperm donors after repeated IUI failures, a decision that may be influenced by both clinical recommendations and personal perceptions. While concerns about donor compatibility are understandable, our findings indicate that in some cases, switching donors can indeed improve success rates. However, the decision to change donors is sometimes driven by information from online forums and social media, where non-evidence-based advice can shape patient choices [33, 34]. This highlights the importance of evidence-based counseling to guide patients toward informed treatment decisions, ensuring that clinical interventions are based on robust scientific data rather than anecdotal experiences.

### Limitations

This study has several limitations. Its retrospective design introduces the potential for selection bias, and the long timeframe (1992–2020) spans periods of significant advancements in laboratory and clinical techniques. Although adjustments were made to account for temporal variability, residual confounding factors cannot be excluded. Additionally, the study did not directly assess sperm-oocyte interactions or genetic compatibility, which may have provided further insights into the observed improvements with donor replacement. Another limitation is the absence of data on maternal complications. Research has established that changing partners in natural conception is associated with increased preeclampsia risk, suggesting that donor replacement might have safety implications that should be evaluated in future studies.

### Future directions

Prospective studies with standardized protocols are essential to validate these findings. Advanced diagnostic tools, such as hemizona assays, could provide insights into the mechanisms of sperm-oocyte compatibility. Future research should include investigating both the efficacy and safety aspects of donor replacement, including potential maternal and neonatal outcomes. Additionally, future research should explore the psychological and emotional impact of donor replacement, particularly in the context of misinformation online, to enhance patient care.

## Data Availability

Data is available upon reasonable request.
